# Steroid-refractory immune checkpoint inhibitor (ICI) hepatitis and ICI rechallenge: A systematic review and meta-analysis

**DOI:** 10.1097/HC9.0000000000000525

**Published:** 2024-09-18

**Authors:** Soo Young Hwang, Pinghsin Hsieh, Wei Zhang

**Affiliations:** 1 Department of Internal Medicine, University of Maryland Midtown Campus, Baltimore, Maryland, USA; 2 Gastroenterology Unit, Massachusetts General Hospital, Harvard Medical School, Boston, Massachusetts, USA

## Abstract

**Background:**

In recent years, the use of immune checkpoint inhibitors (ICIs) has become a cornerstone in cancer treatment. However, this has also resulted in the emergence of immune-related adverse events, notably ICI hepatitis, posing a significant clinical challenge. While steroids are the primary treatment, there are increasing cases of steroid-refractory ICI hepatitis. Our objective is to investigate the management of ICI hepatitis and its response to steroid treatment.

**Methods:**

PubMed/MEDLINE, EMBASE, and CENTRAL databases were searched in July 2023 based on keywords including ICIs (anti–Programmed cell death protein 1/Programmed Death-Ligand 1, anti–CTLA–4, and anti-LAG3) and hepatitis.

**Results:**

A total of 4358 studies were screened, and 44 studies were included in this systematic review. One thousand eight hundred fifty-six patients with ICI hepatitis were included (grade 1-2: 31.7%, grade 3-4: 56.0%, and unknown: 12.3%) with 1184 patients who received corticosteroid treatment. The duration of treatment and dosage varied considerably across the studies. Mycophenolate mofetil was the predominant agent used in 68 out of 82 cases (82.9%), followed by infliximab and azathioprine. A summary estimate of the proportion of steroid-refractory hepatitis in a random effects model was 16% (95% CI: 11%–23%). An estimated 40% (95% CI: 30%–51%) of patients of all patients with ICI hepatitis were rechallenged with an ICI, and of those rechallenged, there was an estimated 22% (95% CI: 15%–30%) recurrence.

**Conclusions:**

Corticosteroids are the primary treatment for ICI hepatitis, with mycophenolate mofetil used as a secondary option for steroids-refractory cases. Current practices mostly rely on expert consensus, highlighting the need for further research to validate and optimize these treatments, particularly for steroid-resistant cases.

## INTRODUCTION

Immune checkpoint inhibitors (ICIs) have become a cornerstone in cancer treatment, demonstrating lasting efficacy even in patients with metastatic cancer, and are increasingly employed in (neo)adjuvant and maintenance therapy.^1^ However, this has also resulted in the emergence of immune-related adverse events (irAEs), which are strongly associated with but not limited to immune activation associated with antitumor immune responses.^2^ Long-term implications and management for irAEs are essential in improving survival with ICIs.

The liver is one of the frequently involved organs in irAE, along with the skin, gut, endocrine gland, and lungs.^3^ Incidence of ICI hepatitis is around 5%–10% of patients treated with ipilimumab, nivolumab, pembrolizumab as single agents but increases as high as 25%–30% in ipilimumab and nivolumab combination therapy.^4^ Steroids are advised as the initial course of treatment, but there are limitations to the current recommendations as the guidelines are derived largely from expert opinion and case studies.^5^

In this study, we aim to conduct a comprehensive review of the treatment approaches and responses for ICI hepatitis, primarily to steroids and secondary immunosuppressants as needed. We further explore the response with rechallenge with an ICI and the recurrent rate of ICI hepatitis.

## METHODS

### Literature search and eligibility

This study was prospectively registered at PROSPERO (registration number: CRD42023450088) and followed the MOOSE reporting guidelines (Supplemental Table S1, http://links.lww.com/HC9/B40). We searched PubMed/MEDLINE, EMBASE, and Cochrane Central Register of Controlled Trials (CENTRAL) databases in July 2023 based on keywords including currently approved “immune checkpoint inhibitors” (anti–Programmed cell death protein 1 (PD-1)/Programmed Death-Ligand 1 (PD-L1), anti–CTLA–4, and anti-LAG3) and “hepatitis” (complete search strategy provided in Supplemental Table S2, http://links.lww.com/HC9/B40) as keywords by investigator (Soo Young Hwang). Two independent researchers (Soo Young Hwang and Pinghsin Hsieh) reviewed the eligibility of the studies independently, and any disagreement was resolved upon discussion between the 2 researchers. Studies that have a description of steroid usage as a treatment for ICI hepatitis or any other treatment for ICI hepatitis were included. Non-English studies, case reports, meeting abstracts, studies on data that were reported in included studies, and studies with insufficient data were excluded.

### Data extraction

From the eligible studies, we extracted the name of the first author, publication year, country, study design, number of patients with ICI hepatitis, stage of ICI hepatitis, cancer type and stage, ICIs, steroid dosage and duration of treatment, secondary immunosuppressive agents, number of patients who were rechallenged, peak ALT levels, adverse events of steroids, and other irAE. The Newcastle–Ottawa Scale (NOS) was applied to assess the risk of bias in the observational studies.

### ICI hepatitis

In the setting that ICI is the most likely cause of liver injury, Common Terminology Criteria for Adverse Events, Version 5 (CTCAE) defines grade 1 hepatitis as AST/ALT 1–3× the upper limit of normal (ULN) or total bilirubin 1–1.5× ULN, grade 2 hepatitis as AST/ALT >3–5× ULN or total bilirubin >1.5–3× ULN, grade 3 hepatitis as AST/ALT >5–20× ULN or total bilirubin >3–10× ULN, and grade 4 hepatitis as AST/ALT >20× ULN or total bilirubin >10× ULN or hepatic decompensation.^6^

### Statistical analysis

Meta-analysis of proportions was performed based on the number of patients treated with steroids and the number of patients requiring a secondary immunosuppressant as the primary outcome. Secondary outcomes were the proportion of patients with ICI hepatitis who were rechallenged with an ICI and the proportion of ICI hepatitis recurrence. The proportion of each study outcome was calculated using a logit transformation. The random effects model was used to obtain the summary estimates, and the summary results were displayed in forest plots. The Q and Higgins I2 statistics were calculated to evaluate the heterogeneity in the included studies.^7^ Publication bias was visually assessed by plotting effect size against sample size (ie, funnel plot) (Supplemental Figure S1, http://links.lww.com/HC9/B40). We performed additional analyses to further explore the heterogeneity of the study. These included subgroup analysis on the country of origin and tumor type, with a focus on melanoma (Supplemental Figure S4, http://links.lww.com/HC9/B40).

A meta-regression analysis was performed based on the primary outcome with moderators, including the percentage of patients who received combination treatment and the percentage of patients with advanced hepatitis (grade 3-4). In addition, we conducted a meta-regression analysis based on the primary outcome and the year of publication. In addition, the association between the number of patients with ICI hepatitis who did not receive any intervention and the percentage of grade 1-2 hepatitis was investigated through a meta-regression analysis.

## RESULTS

**FIGURE 1:**
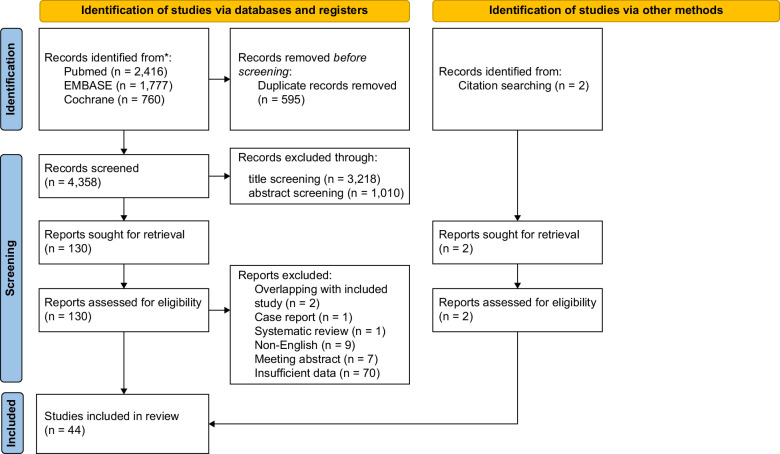
Flow diagram of all included studies. *Consider, if feasible to do so, reporting the number of records identified from each database or register searched (rather than the total number across all databases/registers). **If automation tools were used, indicate how many records were excluded by a human and how many were excluded by automation tools. From Page et al.^52^ For more information, visit: http://www.prisma-statement.org/.

Through a comprehensive search of the 3 databases, 4358 potentially eligible studies were identified and independently screened with an in-depth full-text screening of 130 studies and 44 studies included for final analysis^8–51^ (Figure 1; Tables 1–3).

**TABLE 1:** Basic characteristics of all included studies

			Cancer				Grade of ICI hepatitis				
Study	Age	Male, n (%)	Type (n)	Stage (%)	ICI	N. received ICI	1	2	3	4	Total
Leroy et al^8^	82 [80–90]	14 (60.9)	Melanoma (23)	Stage 4	Ipilimumab	23	0	0	2	0	2
Luo et al^9^			Lung			51					6
Romanski et al^10^	60 [38–87]	19 (44.2)	Melanoma	Stage 4	Ipilimumab (14), pembrolizumab (16), nivolumab (1), ipilimumab + nivolumab (12)	521	179	15	23	5	265
Miller et al^11^	60 [IQR: 54–69]	61 (61)	Melanoma (53), GU (14), lung, head, neck (12), GI (9), other solid (2), hematological (10)	Stage 3 (9) Stage 4 (91)	CTLA-4 monotherapy (25), PD-1/PD-L1 monotherapy (46), combination (29)	5762	0	0	85	15	100
Smith et al^12^	53.8 [IQR: 46.9–60.7]	22 (69)	Melanoma	Stage 3 (8) Stage 4 (92)	Ipilimumab + nivolumab	63	11		21		32
Yamamoto et al^13^	70 [30–84]	14 (66.67)	NSCLC (3), RCC (7), urothelial (1), MM (8), other (2)		Nivolumab (10), pembrolizumab (3), atezolizumab (1), ipilimumab (2), ipilimumab + nivolumab (5)	245	0	7	9	5	21
Takinami et al^14^	55.5 [IQR: 54–68]	4 (50)	Melanoma (6), renal cell (2)		Pembrolizumab (1), ipilimumab (2), ipilimumab + nivolumab (5)	530	0	3	5		8
Owen et al^15^			Melanoma	Stage 4	Anti-PD1, anti-PD1 + anti-CTLA4, anti-PD1 ± anti-CTLA4	118	0	2	8	2	12
Li et al^16^	57.8 (13.7)	47 (54.0)	Melanoma, NSCLC, RCC, breast cancer, urothelial cancer, other		Nivolumab (11), pembrolizumab (43), cemiplimab, ipilimumab (18), ipilimumab + nivolumab (49), anti–PD-L1 (7)	7046	0	0	60	27	87
	61.6 (15.5)	66 (51.6)			Nivolumab (11), pembrolizumab (14), cemiplimab (2), ipilimumab (9), ipilimumab + nivolumab (45), anti–PD-L1 (6)		0	0	106	22	128
Cunningham et al^17^	47.9 (95% CI: 39.3–58.4)	9 (52.9)	Head and neck (4), melanoma (8), pancreas (1), colorectal (2), sarcoma (1), RCC (1)		Anti-PD1 (11), anti–PD-L1 (1), anti-CTLA4 (3), combination (1), blinded (1)	450	0	4	13		17
Sanz-Segura et al^18^						132	2	2	0	0	4
da Silva et al^19^	65	2 (66.7)	Lung (2), melanoma (1)		Pembrolizumab (1), nivolumab (2)	151					3
Huffman et al^20^	57 [32–82]	12 (75)	Melanoma	Stage 4	Ipilimumab (12), pembrolizumab (3), ipilimumab + nivolumab (2)	218	3	1	8	3	17
Cheung et al^21^	62 [21–76]	11 (52)	Melanoma (17), renal cell (1), non–small cell lung (2), epithelial mesothelioma (1)		ipilimumab, nivolumab, pembrolizumab, ipilimumab + nivolumab, Checkmate 238	453	3	4	9	5	21
Shimomura et al^22^			NSCLC	Stage 4	Anti–PD-1 inhibitors	375	18	10	6	0	34
Swanson et al (2022)	70 [54–86]	1 (50)	cSCC		Cemiplimab (2)	39					2
de la Bruyère et al^24^			Melanoma (8), lung (4)		PD(L)-1 inhibitors (6), CTLA-4 inhibitors (6)	150	0	0	12		12
Swanson et al (2022)	65 [47–70]	3 (50)	Pancreatic (3), HCC (2), RCC (1)	Stage 4	Durvalumab combination (6)	112	0	3	3	0	6
Sawada et al^26^	64.0 [48–76]	7 (87.5)	NSCLC (3), MM (1), GC (2), RCC (1), HNSCC (1)		Nivolumab (8), pembrolizumab (5), ipilimumab (4)	135	0	3	5	0	8
Fan et al^27^	60 [IQR: 57–65]	8 (38)	Bladder (2), breast (4), esophageal (2), GBM (2), gastric (2), liposarcoma (1), melanoma (3), NSCLC (3), ovarian (1), pancreatic (1)	Stage 4 (33)	CTLA-4 (20), CTLA-4 + PD-1/PD-L1 (3), PD-1/PD-L1 (16)	331	6		15		21
Kitagataya et al^28^	67 [25–92]	9 (52.9)	Melanoma (5), lung (1), lymphoma (1), other (1)		Nivolumab (8), pembrolizumab (5), ipilimumab (4)	202	3	6	6	2	17
Zheng et al^29^					Anti–PD-1/PD-L1 inhibitor	240	1	0	3	0	4
Daniello et al^30^			NSCLC	Stage 4	Anti-PD(L)1 inhibitors	894	2	7	20	4	33
Cheng et al^31^	63 [56–69]	3 (100)	Melanoma	Stage 4	Ipilimumab						3
Pollack et al^32^			Melanoma	Stage 4	anti–PD-1 + ipilimumab		13		24		37
De Martine et al^33^	63 [33–84]	7 (44)	Melanoma (12), bronchial (1), renal clear cell (1), bladder (1), cholangiocarcinoma (1)	Stage 4	Anti–PD-1/PD-L1 (9), anti-CTLA4 (7)	536	0	0	16		16
Simonaggio et al^34^						159	0	4	8	5	17
Imoto et al^35^	63 [49–69]	31 (63.6)				387	45		11		56
Zen et al^36^	70 [59–74]	8 (80)	NSCLC (4), urothelial (3), merkel cell (1), melanoma (1), colon (1)	Stage 4	Pembrolizumab (6), atezolizumab (4)						10
Riveiro-Barciela et al^37^	62.8 [IQR: 56.6–70.5]	14 (50)	NSCLC (21.4%), melanoma (17.9%), urothelial (14.3%)		Anti-CTLA4 (10), anti–PD-1/PD-L1 (18)	414	0	0	28		28
Gauci et al^38^	52 [IQR: 47–67]	14 (66.7)	Melanoma	Stage 3 (5), Stage 4 (95)	Ipilimumab (7), nivolumab (3), pembrolizumab (1), ipilimumab + nivolumab (10)	339	0	0	10	11	21
Patrinely, Jr. et al^39^	63	88 (53.7)	Lung (12), melanoma (138), renal (5), squamous cell (2), other (7)	Stage 4 (86)	Ipilimumab (7), ipilimumab + nivolumab (97), ipilimumab + pembrolizumab (3), nivolumab (19), pembrolizumab (34), other anti–PD-1/PD-L1 (4)	164	16	50	75	23	164
Rini et al^40^			RCC	Stage 4	Pembrolizumab + axitinib (429), sunitinib (425)	861					125
Lin et al^41^		34 (66.67)			Anti-PD1	1310	37		14		51
Personeni et al^42^	71 [49–83]	5 (55.56)	HCC	BFTABLE CLC stage B or C	Anti–PD-1/PD-L1 ± anti–CTLA–4 antibodies and/or targeted agents (including sorafenib, cabozantinib, and an investigational c-Met inhibitor)	58	0	0	9	0	9
Purde et al^43^	61 [41–73]	6 (54.55)	NSCLC (6), melanoma (5)	Stage 4	Anti-PD1 (6), CTLA4 (1), anti-PD1 + CTLA4 (3)	135	6		4	1	11
Ng et al^44^			HCC	Stage 4		168	12		12		24
Riveiro-Barciela et al^45^	65 [IQR: 56–75]	11 (47.8)	NSCLC (7), Urinary tract (6), melanoma, (4), endometrial (2), HCC (1), cholangiocarcinoma (1), breast(1) chordoma (1)	Stage 3 (30%) Stage 4 (70%)	Anti-PD1 or anti–PD-1/PD-L1 (18), anti–CTLA-4 ± anti-PD1 (4), CD40 agonist antibodies (1)		0	0	19	4	23
Alomari et al^46^				Stage 4	Nivolumab (9), pembrolizumab (7), ipilimumab (1), avelumab (2), nivolumab and ipilimumab (4)	567	8	9	4	2	23
Miah et al^47^	60 [IQR: 51.9–66.8]	30 (46.9)	Head and neck (2), melanoma (24), NSCLC + SCLC (9), RCC (7), Other (22)	Stage 4	PD1 or CTLA monotherapy (46), Combination PD-1 and CTLA-4 (13), other (5)	1096	30		34		64
Hountondji et al^48^	63 [23–89]	63 (53.8)	Melanoma (49), lung (32), renal (16), urothelial (6), cutaneous and oral SCC (7), GI (3), HCC (2), hematological (1), pancreatic(1)	Stage 1-2 (29%) Stage 3 (16%) Stage 4 (54%)	Anti–PD-1 (62), anti–PD-L1 (8), anti–CTLA–4 (4), anti–PD-1 + anti–CTLA–4 (42), anti–PD-1 + anti-LAG-3 (1)	1058	4	17	73	23	117
Matsukane et al^49^						1008	17	15	33		65
Parlati et al^50^	62 [IQR: 48–73]	14 (40)	Melanoma (19), lymphoma (1), NSCLC (10), other (5)		Anti–PD-1 monotherapy (26), anti-PD1/anti-CTLA4 (9)		5	7	12	11	35
Storm et al^51^	62.1 (16.7)	55 (56.7)	Head and neck (10), lung (13), skin (42), GI (5), GU (22), sarcoma (4), other (1)		Pembrolizumab (30), nivolumab (13), ipilimumab/nivolumab combination (43), cemiplimab (2), ipilimumab (5), atezolizumab (4)	2611		37	46	14	97

Age is summarized in median (range), median [IQR Q1-Q3], mean (SD).

Abbreviations: cSCC, cutaneous squamous cell carcinoma; GBM, glioblastoma multiforme; GI, gastrointestinal; GU, genitourinary; HNSCC, head and neck squamous cell carcinoma; ICI, immune checkpoint inhibitor; MM, multiple myeloma; PD-1, programmed cell death protein 1; PD-L1, programmed death-ligand 1; RCC, renal cell carcinoma; NSCLC, non-small cell lung cancer.

**TABLE 2:** Studies on steroid-refractory hepatitis (primary outcome: usage of second-line immunosuppressants)

Study	Total no. ICI hepatitis	No. received steroids	Steroid dose, duration	Side effects of steroid	Peak ALT levels, IU/L	Unit
Romanski et al^10^	265	31	Cumulative dose of prednisolone (mg) grade 2: 737.5 (375–6000) grade 3: 2325 (575–5987.5) grade 4: 4975 (1867.5–6000)			Median (range)
Miller et al^11^	100	67	grade 3: 44 (25–71) days grade 4: 90 (43–121) d		Anti–CTLA–4: 670 (310–2,574), anti–PD-1/PD-L1 482 (297–2946), combination 414 (300–2991)	Median (IQR)
Smith et al^12^	32	31	Induction: mean 69 (23) (mg) prednisone—equivalent/d (adjusted for weight, mean dose of 0.86 mg/kg 0.21 mg/kg)			Mean (SD)
Yamamoto et al^13^	21	13	CS 1 mg/kg (5), 0.7 mg/kg (2), 0.5 mg/kg (2), pulse (5) 10 mg (1)			
Owen et al^15^	12	10	1.8 (1.0–11.4) mo			Median (range)
Li et al^16^	87	87	Initial mPSL ≥1.5 mg/kg maximum CS dose 2.0 (2.0–2.0) i.v. steroids 80 (92.0%) 60 (40–85) d until achieving a prednisone dose ≤10 mg	Infection 16 (18.4%), GI bleed 2 (2.3%), hyperglycemia requiring Tx 20 (23.3%), peak glucose 195 (154–286)	391 (248–606)	Median (IQR)
Li et al^16^	128	128	Initial mPSL <1.5 mg/kg maximum steroid dose 1.0 (1.0–1.3) i.v. steroids 42 (32.8%) 44 (32–70) d until achieving a prednisone dose ≤10 mg	infection 9 (7.0%), GI bleed 3 (2.3%), hyperglycemia requiring Tx 10 (7.8%), peak glucose 166 (137–205)	314 (234–468)	Median (IQR)
Cunningham et al^17^	17	15	DXA 4 mg (1), steroid 1.5 mg/kg i.v. (1), PDN 1 mg/kg (7), PDN taper (2), CS 2 mg/kg i.v. (3) NA		217 (145–324)	Mean (95% CI)
Sanz-Segura et al^18^	4	2	Oral CS 1 mg/kg/d			
Huffman et al^20^	17	16	Prednisone (14), dexamethasone (2), high-dose methylprednisolone (3) 42 (7–78) d		261 (IQR: 110–615)	Median (range) Median (IQR)
Cheung et al^21^	21	18	Dexamethasone (1), prednisolone (11), methylprednisolone (7)		610 (183–1088.5)	Median (IQR)
Shimomura et al^22^	34	7	High-dose (≥0.5 mg/kg of prednisolone) (6), low-dose (<0.5 mg/kg of prednisolone) (1)			
Swanson et al^23^	2	1	6 wk			
de la Bruyère et al^24^	12	7	CS 1 mg/kg (3), ≥2 mg/kg (4) 42 (30–44) d			Median (IQR)
Swanson et al^25^	6	3	CS 1 mg/kg (5), 0.7 mg/kg (2), 0.5 mg/kg (2), pulse (5) 10 mg (1) 28–77 d		415 [30–946]	Median (range)
Fan et al^27^	21	17	Prednisone >1 mg/kg/d: 9 58 (14–111) d	Hyperglycemia (14, 82%), leukocytosis (7, 41%), infection (3, 18%), AMS, melena, venous thromboembolism		Median (IQR)
Kitagataya et al^28^	17	4	PSL 2 mg/kg/d (2), 1 mg/kg/d (1), 1000 mg (1)		185.5 (61–2488)	Median (range)
Zheng et al^29^	4	3	mPSL 2 mg/kg, i.v. 3 d			
Daniello et al^30^	33	27	Initial dose: 87 (92), average dose: 47 (37) 33 (27) d			Mean (SD)
Cheng et al^31^	3	3	mPSL 1 g		372, 1211, 896	
De Martine et al^33^	16	10	Corticosteroid 0.2 mg/kg/d (2), 0.5 mg/kg/d (2), 1 mg/kg/d (5), 2.5 mg/kg/d (1)		460 (266–3137)	Median (range)
Imoto et al^35^	56	4	mPSL 1000 mg/d (1), PSL 0.6 mg/kg/d (2), PSL 1 mg/kg/d (2)		58 (47–129)	Median (range)
Zen et al^36^	10	10	PSL (50 mg/d) (3), PSL (40 mg/d) (3), predonisone (80 mg/d) (1), steroid mini pulse (mPSL, 500 mg/d, 3 d), followed by PSL (50 mg/d) (1), mPSL (1), PSL (1)		226 (93–504)	Median (IQR)
Riveiro-Barciela et al^37^	28	28	Initial dose 60 (52–70) mg/d 2.3 (1.3–3.1) mo	Infection (2)	351 (208–910)	Median (IQR)
Gauci et al^38^	21	13	1 [IQR: 1; 1] (0.3; 2) mg/kg/d 1.8 [IQR: 1.7; 3.5] (1.2–12.6) mo		663 [IQR: 422; 1380] (173–3537)	Median [IQR] (range)
Patrinely, Jr. et al^39^	164	150	PDN or mPSL (147), DXA (1), hydrocortisone (2)| Initially required low-dose steroids (<50 mg daily or <1 mg/kg) (20), required high-dose steroids (129)	Adrenal insuff (2), infection (7), GI (3), hyperglycemia/diabetes (22), insomnia (7), mood changes (7), muscle weakness/myalgias (3), osteoporosis (2), weight gain (3), others (6)		
Rini et al^40^	125	68	High-dose (≥ 40 mg/d of prednisone or equivalent) (61), low-dose (7)			
Lin et al^41^	51	8	Prednisone 0.5–2 mg/kg 3–6 wk			
Personeni et al^42^	9	3	Prednisone 1–2 mg/kg		Grade 3-4: 88 (13 –147) grade 1-2: 37 (11–146)	Median (range)
Purde et al^43^	11	6	80 (13–145) days		NA	Median (IQR)
Riveiro-Barciela et al^45^	23	19	Prednisone (12), methylprednisone (7) recurrence (n = 8) 63 (25) non-recurrence (n = 15) 66 (18) median (range) 8 wk (0.5–51 wk)		280 (188–438)	Median (IQR) mean (SD)
Alomari et al^46^	23	20	>4 wk (18) < 4 wk (2)			
Miah et al^47^	64	46	PDN (23), DXA (6), mPSL (4) median 45 d (range: 21–120 d)			
Matsukane et al^49^	65	29	Low-dose (< 0.5 mg/kg PSL) (n = 93), moderate to high dose (0.5–2.0 mg/kg PSL) (n = 36), i.v. mPSL pulse therapy (500–1000 mg, 3 d) (n = 41)			

Abbreviations: CS, corticosteroid; DXA, dexamethasone; mPSL, methylprednisolone; PD-1, programmed cell death protein 1; PD-L1, programmed death-ligand 1; PDN, prednisone; PSL, prednisolone.

**TABLE 3:** Studies on recurrence of immune checkpoint inhibitor hepatitis

Study	No. treated with steroids	No. treated with secondary immunosuppressants	No. rechallenged/recurrence
Leroy et al^8^	2	1 (MMF)	
Luo et al^9^	6	5 (MMF)	1/0
Romanski et al^10^	31	2 (MMF)	
Miller et al^11^	67	3 (MMF)	31/8
Smith et al^12^	31	1 (infliximab)	17/3
Yamamoto et al^13^	13	2 (MMF)	
Takinami et al^14^	6	2 (MMF)	3/0
Owen et al^15^	10	1 (MMF, azathioprine)	
Li et al^16^	87	32	
Li et al^16^	128	29	
Cunningham et al^17^	15	1 (MMF)	7/1
da Silva et al^19^	3		1/0
Huffman et al^20^	16	2 (AZA 1 CsA 1)	
Cheung et al^21^	18	10 (infliximab 2 MMF 8 tacrolimus 1)	4/0
de la Bruyère et al^24^	7	1	3/1
Swanson et al (2022)	3	0	1/0
Fan et al^27^	17	6 (MMF)	
Kitagataya et al^28^	4	2 (MMF)	
Zheng et al^29^	3	1 (MMF, gamma globulin)	
Daniello et al^30^	27	2	
Cheng et al^31^	3	0	
Pollack et al^32^	36	3 (MMF)	29/5
De Martine et al^33^	10	1 (MMF)	3/1
Simonaggio et al^34^	13	2 (MMF)	5/3
Imoto et al^35^	4	3 (MMF 2, infliximab 1)	
Zen et al^36^	10	1 (MMF, AZA)	
Riveiro-Barciela et al^37^	28	10	6/0
Gauci et al^38^	13	0	8/0
Patrinely, Jr. et al^39^	150	37	66/17
Rini et al^40^	68		100/45
Personeni et al^42^	3		6/0
Purde et al^43^	6	0	3/1
Riveiro-Barciela et al^45^	19	2 (MMF)	23/8
Miah et al^47^	46	3 (MMF, MMF+infliximab)	11/0
Hountondji et al^48^	93	18 (MMF 17 rituximab 1)	51/12
Matsukane et al^49^	29		33/8
Parlati et al^50^	20		8/0
Storm et al^51^	78	10 (MMF 9, other 1)	32/13
Cunningham et al^17^	15	1 (MMF)	7/1
da Silva et al^19^	3		1/0
Huffman et al^20^	16	2 (AZA 1 CsA 1)	
Cheung et al^21^	18	10 (infliximab 2 MMF 8 tacrolimus 1)	4/0
de la Bruyère et al^24^	7	1	3/1
Swanson et al (2022)	3	0	1/0
Fan et al^27^	17	6 (MMF)	
Kitagataya et al^28^	4	2 (MMF)	
Zheng et al^29^	3	1 (MMF, gamma globulin)	
Daniello et al^30^	27	2	
Cheng et al^31^	3	0	
Pollack et al^32^	36	3 (MMF)	29/5
De Martine et al^33^	10	1 (MMF)	3/1
Simonaggio et al^34^	13	2 (MMF)	5/3
Imoto et al^35^	4	3 (MMF 2, infliximab 1)	
Zen et al^36^	10	1 (MMF, AZA)	
Riveiro-Barciela et al^37^	28	10	6/0
Gauci et al^38^	13	0	8/0
Patrinely, Jr. et al^39^	150	37	66/17
Rini et al^40^	68		100/45
Personeni et al^42^	3		6/0
Purde et al^43^	6	0	3/1
Riveiro-Barciela et al^45^	19	2 (MMF)	23/8
Miah et al^47^	46	3 (MMF, MMF + infliximab)	11/0
Hountondji et al^48^	93	18 (MMF 17 rituximab 1)	51/12
Matsukane et al^49^	29		33/8
Parlati et al^50^	20		8/0
Storm et al^51^	78	10 (MMF 9, other 1)	32/13

Abbreviations: AZA, azathioprine; CsA, cyclosporine; MMF, mycophenolate mofetil.

### Baseline characteristics

A total of 1856 patients with ICI hepatitis were included. Five hundred ninety (31.7%) of the patients developed grade 1-2 hepatitis, and 1,043 (56.0%) of the patients developed grade 3-4 hepatitis.

The prevalence of ICI hepatitis in our study was 6.38% (1856 cases out of 29,112 patients who received an ICI). The estimated median age of patients with ICI hepatitis was 63 (range: 21–90), with 55.7% (692 out of 1243) of male patients in advanced stages of cancer, stages 3 and 4. Ten studies were conducted in Asia, 13 studies were conducted in North America, 17 studies in Europe, 1 in Australia, and 3 studies were multinational. ICI included in the study were anti-PD1 nivolumab, pembrolizumab, cemiplimab; anti–PD-L1 atezolizumab, durvalumab; and anti-CTLA4 ipilimumab. Combination therapies consist of ipilimumab and nivolumab, ipilimumab and pembrolizumab. 37.52% (454 out of 1218) of patients were treated with combination therapy, and 62.48% (756 out of 1218) of patients were treated with monotherapy. Two hundred eighteen (38.05%) of the patients experienced disease progression regarding ICI, while 355 (61.95%) of the patients experienced stable disease or response from the ICI.

### Steroid as a first-line treatment of ICI hepatitis

**FIGURE 2:**
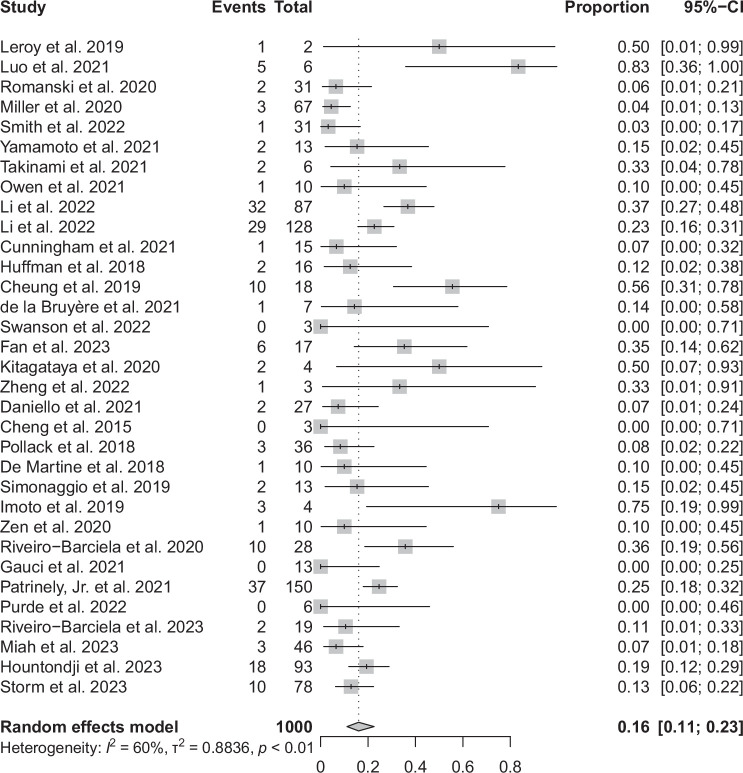
Forest plot of the proportion of steroid-refractory ICI hepatitis. Abbreviation: ICI, immune checkpoint inhibitor.

One thousand one hundred eighty-four patients out of a total of 1864 patients received corticosteroid treatment Table 2. The duration of treatment varied considerably across the studies, ranging from 3 to 361 days. Similarly, there was substantial variation in dosage, from oral prednisone at 0.5 mg/kg to i.v. methylprednisolone at 2 mg/kg. In total, 32 studies reported on steroid-refractory cases that necessitated the use of second-line immunosuppressants. Mycophenolate mofetil was the predominant agent used in 68 out of 82 cases (82.9%). Other treatments included infliximab in 5 out of 82 cases (6.1%), azathioprine in 3 out of 82 cases (3.7%), and 1 case each for rituximab, gamma globulin, tacrolimus, and cyclosporine. A summary estimate of the proportion of steroid-refractory hepatitis in a random effects model was 16% (95% CI: 11%–23%) (Figure 2). There was moderate heterogeneity (*I* ^2^ = 60%) in the analysis. The funnel plot (Supplemental Figure S1, http://links.lww.com/HC9/B40) showed no visual asymmetry, and statistical analysis showed no evidence of publication bias (*p* < 0.001). Subgroup analyses based on the country of origin did not demonstrate statistically significant differences in the proportion of patients requiring additional immunosuppressants (chi-square 5.71, df = 3, *p* = 0.13) (Supplemental Figure S3, http://links.lww.com/HC9/B40) and there was no statistically significant association with the publication year (coefficient = −0.031, *p* = 0.784) (Supplemental Figure S5, http://links.lww.com/HC9/B40).

The proportion of patients requiring additional immunosuppressants was not statistically associated with percentage of combination ICI therapy (coefficient = −0.461, *p =* 0.546) or percentage of grade 3-4 hepatitis (coefficient = 0.03, *p =* 0.976).

An estimated 23% (95% CI: 15%–35%) of the patients with ICI hepatitis did not receive any steroids, correlated with the proportion of grade 1-2 hepatitis in the cohort (coefficient = 3.22, *p <* 0.001) (Supplemental Figure S2, http://links.lww.com/HC9/B40). The most common side effects of steroid treatment were infection (11.6%, 38 out of 329 cases) and hyperglycemia (20.1%, 66 out of 329 cases). Other side effects reported were gastrointestinal bleeding, altered mental status, mood changes, muscle weakness or myalgia, and osteoporosis.

### ICI rechallenge

**FIGURE 3:**
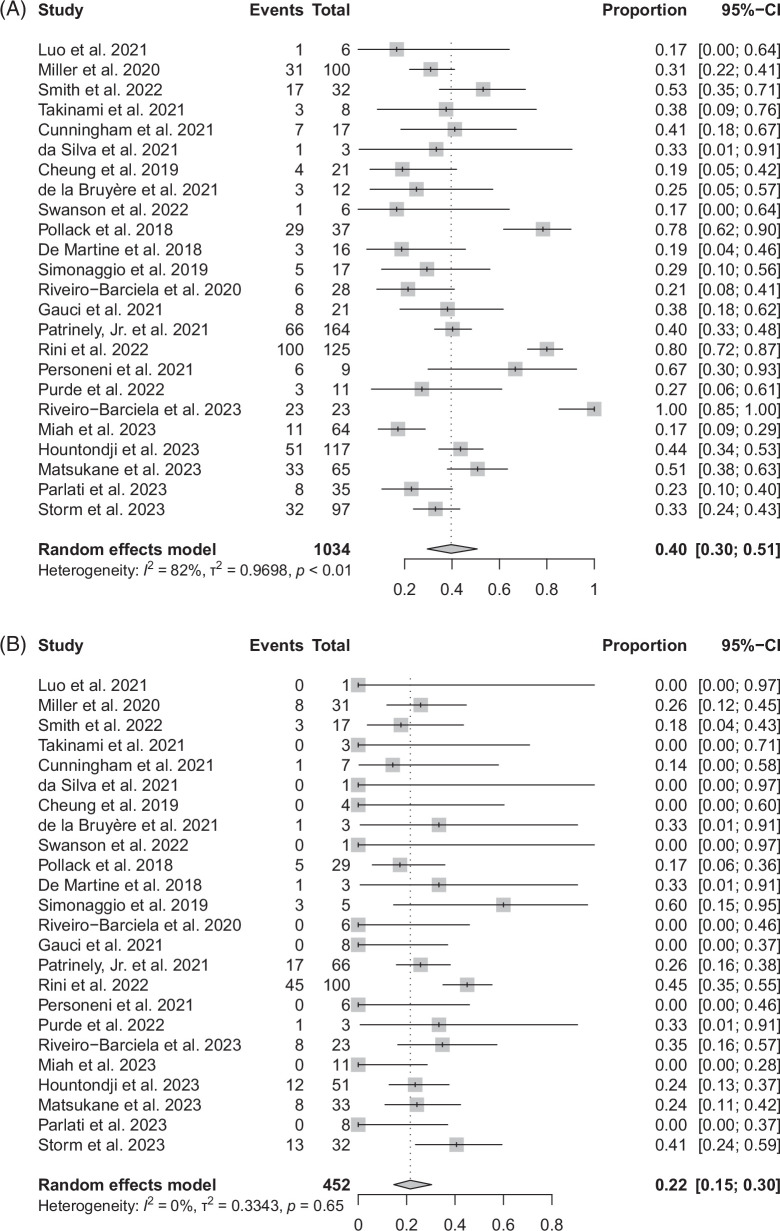
(A) Proportion of patients who were rechallenged with an ICI. (B) Proportion of ICI recurrences in patients who were rechallenged. Abbreviation: ICI, immune checkpoint inhibitor.

After the resolution of ICI hepatitis or improvement to grade 1 hepatitis, patients were rechallenged with the ICI based on clinical judgment Table 3. An estimated 40% (95% CI: 30%–51%) of patients of all patients with ICI hepatitis were rechallenged with an ICI, and of those rechallenged (Figure 3A), there was an estimated 22% (95% CI: 15%–30%) recurrence (Figure 3B). There was high heterogeneity (*I* ^2^ = 81.8%) in the proportion of patients rechallenged out of the total patients with ICI hepatitis. The funnel plot analysis showed no evidence of publication bias (*p* < 0.001) for this outcome. Previously developing advanced ICI hepatitis (grade 3-4) did not have a significant association with the proportion of patients rechallenged (coefficient = 0.197, *p* = 0.848) nor the recurrence of ICI hepatitis (coefficient = 0.449, *p* = 0.553).

## DISCUSSION

Steroid treatment was the primary intervention in over 75% of patients with ICI hepatitis while 16% of the patients who received steroids required a secondary immunosuppressant in management. An estimated 23% of patients, mostly with grades 1-2 hepatitis, did not require any intervention. Of those who were rechallenged with an ICI, only 22% of the patients experienced a recurrence of ICI hepatitis. Steroids are the treatment of choice given that it is considered that high-dose glucocorticoids do not interfere with the antitumor response of ICIs but there are also controversial studies against this.^1,53^

Current AGA guidelines suggest liver monitoring for grade 1 hepatitis, ICI discontinuation for grade 2 and higher, and if the patient is symptomatic of liver toxicity, an equivalent of prednisone 0.5–1.0 mg/kg/d should be administered for grade 2 hepatitis. For grade 3 hepatitis, initiation of an equivalent of 1–2 mg/kg of methylprednisone is recommended, and a second-line immunomodulator such as an azathioprine or mycophenolate mofetil can be considered if there is no clinical improvement in 3–5 days. For grade 4 hepatitis, permanent discontinuation of ICI and initiation of an equivalent of 2 mg/kg/d of methylprednisone is recommended.^5^ Third-line immunosuppressive therapy brought into consideration is anti-thymocyte globulin for ipilimumab-induced hepatitis or tacrolimus, whereas infliximab is not recommended.^4^

Several studies included in our analysis asserted that there is greater risk than benefit in the use of high-dose steroids compared to low-dose steroids and association with poor survival.^9,16,22,27,49^ This can be interpreted by 3 hypotheses: (1) patients who are treated with high-dose steroids have more advanced hepatitis; (2) patients with advanced cancer treated with ICIs are at higher risk for side effects of immunosuppression, especially infection; and (3) high-dose steroids compromise the effectiveness of ICIs. Li et al^16^ compared 87 patients in the ≥1.5 mg/kg methylprednisone equivalent group and 128 patients in the <1.5 mg/kg group with grade 3-4 ICI hepatitis and reported that there was no difference in the development of steroid-refractory hepatitis but longer exposure and higher incidence of infection. However, the high-dose steroid group also had a higher percentage of ipilimumab and nivolumab combination therapy, which can contribute to a higher risk of disease.^16^ Corticosteroids can inhibit the antitumor immune response of ICIs by suppressing low-affinity memory T cells, particularly in a higher dose and earlier administration timing.^54^

Anti–CTLA–4 mAbs have been associated with a higher incidence of ICI hepatitis compared to anti-PD1/anti–PD-L1 mAbs, and combination therapy was considered a higher risk than monotherapy, although our study did not demonstrate a statistically significant relationship.^10,17,47^ Several studies have suggested that specific histopathologic patterns may correlate with the type of ICI used. Furthermore, these studies indicate that treatment responses may vary based on the characteristic histopathologic pattern of ICI hepatitis. De Martin et al^33^ observed a more prevalent pattern of granulomatous hepatitis with anti–CTLA–4 mAbs and a more heterogeneous pattern, mainly lobular hepatitis in anti–PD-1/PD-L1 mAbs. Different histopathologic patterns were also associated with different treatment responses. A study of 20 biopsied patients reported that patients with an acute granulomatous profile defined by the presence of granulomas or acute hepatitis with a toxic profile defined by the presence of eosinophilic polynuclear cells had a better response to corticosteroids, whereas patients with a cholangitic lesion with recorded bile duct lesions had a worse response.^50^

As a second-line immunomodulator, mycophenolate mofetil was used in the majority of cases refractory to steroids. Interestingly, infliximab, which was not recommended in the AGA guidelines due to potential idiosyncratic liver injury, was the second-line drug of choice in 5 cases and azathioprine in 3 cases.^5^ Mycophenolate mofetil is a purine antagonist that inhibits the proliferation and activation of both T and B lymphocytes and has been used as a second-line agent for steroid-refractory autoimmune hepatitis.^55–57^ Azathioprine, traditionally the first-line steroid-sparing agent for autoimmune hepatitis, is less favored in ICI treatment. This is due to its slow onset of immunosuppressive effect, which can take several months to reach peak efficacy. In addition, azathioprine’s metabolites can potentially cause hepatotoxicity, further complicating its use in patients already experiencing liver inflammation.^31,58^ While the selection of second-line immunomodulators originates from agents used to manage autoimmune hepatitis, it is worth noting that ICI hepatitis exhibits distinct characteristics compared to autoimmune hepatitis, including analytic factors such as lower levels of gammaglobulins, immunoglobulin G, and ANAs.^37^

Diagnosis and management of ICI hepatitis are challenging in that it is a distinct etiology that is a DILI but also has components of immunological characteristics. ICI hepatitis is a clinical diagnosis of exclusion, and certain adjunctive parameters, such as the RUCAM score, were used to assist in determining whether hepatitis is a DILI.^59^ Also, as the majority of studies for ICI hepatitis are conducted on patients with advanced cancer, such as patients with stage 4 melanoma or non–small cell lung cancer, hepatic metastases can be a confounding factor in the evaluation of ICI hepatitis.^10,25^

ICIs were rechallenged after resolution or improvement to grade 1 hepatitis in an estimated 40% of the cases. Recurrence of ICI hepatitis was present in 22% of all rechallenged cases, mainly in anti–PD-1/PD-L1 agents, and was noted to be not as severe as the initial event.^34,45,48^ Hountondji et al^48^ suggested that rechallenge was even possible after grade 3-4 hepatitis. ICI rechallenge is important because patients at advanced cancer stage have limited options for treatment and because irAEs, including ICI hepatitis, have been associated with improved antitumor efficacy and overall survival.^44,46,47,60^ Our findings suggest that rechallenge of ICIs should be reconsidered more frequently after successful treatment of ICI hepatitis. Two studies compared the outcome between patients who underwent ICI rechallenge and those who did not; Simonaggio et al^34^ found no significant difference in median progression-free survival time between the rechallenged and non-rechallenged groups, including irAE from other systems. Similarly, Miah et al^47^ reported no difference in best overall response or time to death between these groups. However, these findings need to be interpreted cautiously due to the potential for substantial selection bias based on the severity and treatment response of ICI hepatitis. It is also critical that rechallenge would often involve a different regimen, such as switching the class from anti-CTLA4 to anti-PD (L)1 therapy or de-escalation from combination therapy to monotherapy.^11,14,21,37,38,48,51^

Our study is the first meta-analysis to quantify the prognosis and treatment response of ICI hepatitis with steroid treatment as the primary treatment. However, our study also had several limitations. First, the variability in the dosage and duration of steroids were high between studies, and it could have been an overgeneralization in estimating the effect of steroids on whether patients received steroid treatment or not. Second, not all studies reported patient characteristics we considered important. For example, while earlier studies provided the detailed dosage and regimen of ICI therapy, most recent studies only included broad categories of ICI therapy used, potentially introducing greater heterogeneity into the analysis. Lastly, although we determined that this is minimal in our study, there is still a possibility of publication bias.

## CONCLUSIONS

Our meta-analysis reveals that corticosteroids remain the primary treatment for ICI hepatitis, with mycophenolate mofetil serving as a secondary option in steroids-refractory cases. ICI rechallenge resulted in recurrence in approximately one-fifth of the cases, typically with less severe presentations. However, current practices largely rely on expert consensus, highlighting the need for prospective studies on key areas. These include establishing standardized steroid treatment protocols, evaluating the efficacy of mycophenolate mofetil in steroid-refractory cases, and assessing the safety and efficacy of ICI rechallenge following ICI hepatitis.

## Supplementary Material
