# Supplementary material for: Steroid-refractory immune checkpoint inhibitor (ICI) hepatitis and ICI rechallenge: A systematic review and meta-analysis
Source: Hepatol Commun. 2024 Sep 18;8(10):e0525. doi: 10.1097/HC9.0000000000000525 (PMC11412713; doi:10.1097/HC9.0000000000000525)
Supplement: Supplementary file 1 [file hc9-8-e0525-s001.docx]

**Supplemental Table 1. MOOSE Guidelines for Meta-Analyses and Systematic Reviews of Observational Studies***

**Title**  Identify the study as a meta-analysis (or systematic review): pg 1.

**Abstract** Use the journal’s structured format: pg 2.

**Introduction Present**

· The clinical problem: pg 3.

· The hypothesis: pg 3.

· A statement of objectives that includes the study population, the condition of interest, the

exposure or intervention, and the outcome(s) considered: pg 3.

**Sources Describe**

· Qualifications of searchers (eg, librarians and investigators): pg 4.

· Search strategy, including time period included in the synthesis and keywords: pg 4. Supplemental Table 2.

· Effort to include all available studies, including contact with authors: Figure 1.

· Databases and registries searched: pg 3.

· Search software used, name and version, including special features used (eg, explosion): not described.

· Use of hand searching (eg, reference lists of obtained articles): Figure 1.

· List of citations located and those excluded, including justification: not described.

· Method of addressing articles published in languages other than English: not described.

· Method of handling abstracts and unpublished studies: pg 4.

· Description of any contact with authors: not described.

**Study Selection Describe**

· Types of study designs considered: pg 4.

· Relevance or appropriateness of studies gathered for assessing the hypothesis to be tested: pg 4.

· Rationale for the selection and coding of data (eg, sound clinical principles or convenience): pg 4.

· Documentation of how data were classified and coded (eg, multiple raters, blinding, and

interrater reliability): pg 4.

· Assessment of confounding (eg, comparability of cases and controls in studies where

appropriate): pg 4.

· Assessment of study quality, including blinding of quality assessors; stratification or

regression on possible predictors of study results: pg 4-5.

· Assessment of heterogeneity: pg 5.

· Statistical methods (eg, complete description of fixed or random effects models, justification of whether the chosen models account for predictors of study results, dose-response models, or cumulative meta-analysis) in sufficient detail to be replicated: pg 5.

**Results Present**

· A graph summarizing individual study estimates and the overall estimate: Figure 2, 3.

· A table giving descriptive information for each included study: Table 1.

· Results of sensitivity testing (eg, subgroup analysis): not applicable.

· Indication of statistical uncertainty of findings: not described.

**Discussion Discuss**

· Strengths and weaknesses: pg 10.

· Potential biases in the review process (eg, publication bias): pg 10.

· Justification for exclusion (eg, exclusion of non–English-language citations): not described.

· Assessment of quality of included studies: not described.

· Consideration of alternative explanations for observed results: pg 8-9.

· Generalization of the conclusions (ie, appropriate for the data presented and within the domain of the literature review): pg 11.

· Guidelines for future research: pg 10.

· Disclosure of funding source: pg 1.

*Modified from Stroup DF, Berlin JA, Morton SC, Olkin I, Williamson GD, Rennie D, et al. Meta-analysis of observational studies in epidemiology: a proposal for reporting. Meta-analysis Of Observational Studies in Epidemiology (MOOSE) group. JAMA 2000;283:2008–12.

**Supplemental Table 2. Full Search Strategy**

Search was performed on July 38, 2023.

| **Search Engine** |
| --- |
| (Immune checkpoint inhibitor OR ICI OR CTLA-4 OR Ipilimumab OR Tremelimumab OR Toripalimab OR Sintilimab OR Anti-PD-1 OR Nivolumab OR Pembrolizumab OR Cemiplimab OR Camrelizumab OR Tislelizumab OR Penpulimab OR Zimberelimab OR Dostarlimab OR Anti-PD-L1 OR Anti-PDL1 OR Atezolizumab OR Avelumab OR Durvalumab OR Envafolimab OR Sugemalimab OR Anti-LAG3 OR Anti-LAG-3 OR Relatlimab) AND (hepatitis OR hepatotoxicity OR hepatic adverse effect OR hepatic failure OR liver toxicity OR liver failure) |
| Result: Pubmed/MEDLINE 2416 hits, EMBASE 1777 hits, Cochrane 760 hits |

**Supplemental Figure 1. Funnel plot of all included studies.**

**Supplemental Figure 2. Forest plot of proportions that did not receive any steroids.**

**Supplemental Figure 3. Forest plot of a subgroup analysis of proportions of steroid-refractory ICI hepatitis based on country of origin.**

**Supplemental Figure 4. Forest plot of a subgroup analysis of proportions of steroid-refractory ICI hepatitis based on melanoma as tumor type.**

**Supplemental Figure 5. Meta-regression of publication year and the proportion of steroid-refractory ICI hepatitis (coefficient= -0.031, p-value = 0.784).**
